# Demographic data for urinary Acute Kidney Injury (AKI) marker [IGFBP7]·[TIMP2] reference range determinations

**DOI:** 10.1016/j.dib.2015.10.036

**Published:** 2015-11-05

**Authors:** Nandkishor S. Chindarkar, Lakhmir S. Chawla, Joely A. Straseski, Saeed A. Jortani, Denise Uettwiller-Geiger, Robert R. Orr, John A. Kellum, Robert L. Fitzgerald

**Affiliations:** a Department of Pathology, Center for Advanced Laboratory Medicine, University of California, San Diego Health Systems, San Diego, CA, USA; b Department of Anesthesiology and Critical Care Medicine, George Washington, University Medical Center, Washington, DC, USA; c Department of Pathology and ARUP Laboratories, University of Utah School of Medicine, Salt Lake City, UT, USA; d Department of Pathology, University of Louisville, Louisville, KY, USA; e John T. Mather Memorial Hospital, Port Jefferson, NY, USA; f Phoenix Medical Group, Peoria, AZ, USA; g Center for Critical Care Nephrology Department of Critical Care Medicine, University of Pittsburgh, Pittsburgh, PA, USA

**Keywords:** Reference ranges, Acute kidney injury, Biomarkers, IGFBP7, Insulin-like growth factor-binding protein 7, TIMP2, Tissue inhibitor of metalloproteinases-2

## Abstract

This data in brief describes characteristics of chronic stable comorbid patients who were included in reference range studies of [IGFBP7]·[TIMP-2] “Reference Intervals of Urinary Acute Kidney Injury (AKI) Markers [IGFBP7]·[TIMP2] in Apparently Healthy Subjects and Chronic Comorbid Subjects without AKI” [1]. In order to determine the specificity of [IGFBP7]·[TIMP-2] for identifying patients at risk of developing AKI we studied a cohort with nine broad classification of disease who did not have AKI. Details regarding the population that was targeted for inclusion in the study are also described. Finally, we present data on the inclusion criteria for the healthy subjects used in this investigation to determine the reference range.

## **Specifications table**

**Table:** 

Subject area	*Healthcare*
More specific subject area	*Acute kidney injury*
Type of data	*Tables*
How data was acquired	*List of patient characteristics*
Data format	*Tables*
Experimental factors	*Healthy subjects and chronic comorbid subjects without acute kidney injury*
Experimental features	*Design of experiments reflect US patient characteristics*
Data source location	*Subjects recruited from Rochester, NY; Dallas, TX; Gresham, OR; Springfield, MO; Layton, UT; Peoria, AZ*
Data accessibility	*Data are with this article*

## **Value of the data**

• The data described allow other researchers to understand the patient cohort we used to determine the specificity of the AKI biomarkers [IGFBP7]·[TIMP-2] in the setting of stable chronic comorbid conditions.
• We included patients with cardiovascular, respiratory, gastrointestinal, renal, muscular skeletal, endocrine, and neuromuscular disease in the stable chronic comorbid condition cohort who did not have AKI, which serves as a model for future studies.
• We describe inclusion criteria for a healthy reference range population that can also be used for future studies evaluating biomarkers of AKI.

## 1 Data

The data described provide details on the conditions and numbers of subjects evaluated who did not have AKI but did have other chronic stable comorbid conditions that were used to demonstrate the specificity of these biomarkers for AKI. We also describe the targeted patient population and the inclusion criteria that were used to determine the reference range of [IGFBP7]·[TIMP-2] in healthy individuals.

## 2 Experimental design, materials and methods

The reference range study was designed to include patients commonly seen in intensive care units of hospitals in the United States [2]. The list of patients with chronic stable comorbid conditions is presented in Table 1. Table 2 gives a description of patient demographics that were targeted for inclusion. Table 3 provides detailed inclusion criteria used to select the healthy reference range population.

The protocols for this investigation were approved by investigational review boards/ethics committees as required by each participating institution. All subjects provided written informed consent. Subjects of ≥21 years age, who provided written informed consent for the study participation, and met the morbidity criteria (Table 1) were selected in the stable chronic morbidity cohort. For apparently healthy subjects, individuals of ≥21 years of age, who provided written informed consent for study participation, and met the healthy criteria (Table 2), were selected for this cohort.

The patients were recruited at 6 geographically diverse sites (Rochester, NY; Dallas, TX; Gresham, OR; Springfield, MO; Layton, UT; Peoria, AZ). In the stable chronic comorbid cohort most patients had several comorbidities, with the most prevalent being some type of an endocrine or cardiovascular disorder. In terms of specific comorbidities, as might be expected for the US population, the highest prevalence was hypertension (59.7%) with the other top four being hypercholesterolemia (40.1%), osteoarthritis (28.8%), and diabetes (24.5%).

## Figures and Tables

**Table 1:** Medical conditions for chronic stable morbidity cohort (*N*=372).

Medical condition	*N*	%
Any cardiovascular	237	(63.7)
CAD	27	(7.3)
Bypass graft	2	(0.5)
CHF	12	(3.2)
Hypertension	222	(59.7)
MI	0	(0.0)
Arrhythmia	32	(8.6)
PVD	5	(1.3)
		
Any respiratory	99	(26.6)
Emphysema	10	(2.7)
Sleep apnea	41	(11.0)
COPD	32	(8.6)
Chronic bronchitis	16	(4.3)
Asthma	51	(13.7)
		
Any gastrointestinal	14	(3.8)
Cirrhosis	2	(0.5)
Hepatic failure	0	(0.0)
IBD	5	(1.3)
Peptic ulcers	1	(0.3)
Chronic pancreatitis	0	(0.0)
GI bleeding	1	(0.3)
Crohn׳s disease	1	(0.3)
Ulcerative colitis	4	(1.1)
		
Any renal	13	(3.5)
Renal insufficiency	6	(1.6)
Polycystic kidney disease	1	(0.3)
Nephrolithiasis	4	(1.1)
Other	6	(1.6)
		
Any musculoskeletal	116	(31.2)
Osteoarthritis	107	(28.8)
Gout	15	(4.0)
		
Any endocrine/metabolic	255	(68.5)
Any Diabetes	91	(24.5)
Type I Diabetes	7	(1.9)
Type II Diabetes	84	(22.6)
Hypercholesterolemia	149	(40.1)
Hyperlipidemia	53	(14.2)
Hyperthyroidism	6	(1.6)
Hypothyroidism	69	(18.5)
Metabolic syndrome	1	(0.3)
		
Any neurological	41	(11.0)
Neuromuscular disease	19	(5.1)
Stroke	0	(0.0)
Seizures	3	(0.8)
Migraines	25	(6.7)
		
Any immune disorder	21	(5.6)
Rheumatoid arthritis	16	(4.3)
Immunocompromised	4	(1.1)
Lupus	2	(0.5)
AIDS	0	(0.0)
		
Other conditions	214	(57.5)
Coagulation abnormality	4	(1.1)
Organ transplant	0	(0.0)
Trauma	0	(0.0)
Surgeries	0	(0.0)
BPH	38	(21.0)
Psoriasis	8	(2.2)
Drug Abuse	5	(1.3)
Any Other	189	(50.8)
Cancer	9	(2.4)
Any metastatic cancer	1	(0.3)
Active cancer	5	(1.3)
Cured/In remission	3	(0.8)

CAD, coronary artery disease; CHF, congestive heart failure; MI, myocardial infarction; PVD, peripheral vascular disease; COPD, chronic obstructive pulmonary disease; IBD, inflammatory bowel disease; GI, gastrointestinal; AIDS, acquired immunodeficiency syndrome; and BPH, benign prostatic hypertrophy.

**Table 2:** The following percent distribution for age, race, ethnicity and gender was targeted during selection of participants. This distribution was targeted to reflect the general demographic data for United States intensive care units.

	Percentage of total enrollment (%)
Age range (years)	
<46	12
46-55	16
56–65	20
66–75	26
>75	26
	
Race	
Black	14
White	72
American Indian/Alaskan	1
Other	13
	
Ethnicity	
Hispanic/Latino	9
Not Hispanic Latino	91
	
Gender	
Female	50
Male	50

**Table 3:** List of apparently healthy subject criteria.

A subject was deemed apparently healthy, if he or she was ≥21 years of age, did not have any chronic, stable morbid conditions (see Table 1), and met all following criteria:
1. Subject without any known or suspected acute illness or condition-including acute infections – at the time of enrollment or within the previous 30 days
2. Subject without any new onset or unstable morbidities listed under “Chronic, stable morbid conditions”
3. Subject without any trauma-related surgery within the last 6 months
4. Subject without any surgery, hospitalization or institutionalization (such as in a nursing home) during the previous 3 months
5. Subject did not receive any blood product transfusion within the previous 2 months
6. Subject who was not a pregnant woman or child
7. Subject was not prisoners or institutionalized individual
8. Subject who did not provide evaluable blood or urine samples for this study
